# Raiders of the Lost Bark: Orangutan Foraging Strategies in a Degraded Landscape

**DOI:** 10.1371/journal.pone.0020962

**Published:** 2011-06-22

**Authors:** Gail Campbell-Smith, Miran Campbell-Smith, Ian Singleton, Matthew Linkie

**Affiliations:** 1 Durrell Institute of Conservation and Ecology, University of Kent, Canterbury, Kent, United Kingdom; 2 Human-Orangutan Conflict and Mitigation Programme, Orangutan Information Centre, Medan, North Sumatra, Indonesia; 3 Sumatran Orangutan Conservation Programme, PanEco Foundation, Medan, North Sumatra, Indonesia; 4 Fauna & Flora International, Cambridge, United Kingdom; Smithsonian's National Zoological Park, United States of America

## Abstract

Deforestation is rapidly transforming primary forests across the tropics into human-dominated landscapes. Consequently, conservationists need to understand how different taxa respond and adapt to these changes in order to develop appropriate management strategies. Our two year study seeks to determine how wild Sumatran orangutans (*Pongo abelii*) adapt to living in an isolated agroforest landscape by investigating the sex of crop-raiders related to population demographics, and their temporal variations in feeding behaviour and dietary composition. From focal animal sampling we found that nine identified females raided cultivated fruits more than the four males. Seasonal adaptations were shown through orangutan feeding habits that shifted from being predominantly fruit-based (56% of the total feeding time, then 22% on bark) to the fallback food of bark (44%, then 35% on fruits), when key cultivated resources such as jackfruit (*Artocarpus integer*), were unavailable. Cultivated fruits were mostly consumed in the afternoon and evening, when farmers had returned home. The finding that females take greater crop-raiding risks than males differs from previous human-primate conflict studies, probably because of the low risks associated (as farmers rarely retaliated) and low intraspecific competition between males. Thus, the behavioral ecology of orangutans living in this human-dominated landscape differs markedly from that in primary forest, where orangutans have a strictly wild food diet, even where primary rainforests directly borders farmland. The importance of wild food availability was clearly illustrated in this study with 21% of the total orangutan feeding time being allocated to feeding on cultivated fruits. As forests are increasingly converted to cultivation, humans and orangutans are predicted to come into conflict more frequently. This study reveals orangutan adaptations for coexisting with humans, e.g. changes in temporal foraging patterns, which should be used for guiding the development of specific human-wildlife conflict mitigation strategies to lessen future crop-raiding and conflicts.

## Introduction

Across the humid tropics, widespread deforestation is dramatically changing habitat and food resource compositions through converting primary forest into mosaic landscapes of mixed agriculture interspersed with patches of remnant forests [1]. Arboreal taxa, especially non-human primates (hereafter primates), are particularly sensitive to the disruption of forest canopy integrity [2]. However, the ability of different primate species to adapt to living in agroforest systems may depend on their behaviour, ecology and the types of anthropogenic disturbance to the forest. For example, primates with specialized diets such as leaf monkeys (e.g. proboscis monkeys, *Nasalis larvatus*) may not be able to sufficiently alter their natural diet if patches of forest are replaced by unpalatable crops, such as waxy-leafed coffee plants or tough fibrous oil palms. However, primate species with greater dietary plasticity may supplement their natural diet by raiding highly nutritious crops, as found, for example, in macaques (*Macaca sp.*; [3], [4]) and baboons (*Papio sp.*; [5]).

Changes in the quality and quantity of food sources, both wild and cultivated, may cause animals to trade-off their activity budgets in different ways. Some species, such as olive baboons (*Papio anubis*), may allocate more time to searching for highly nutritious food patches [6], while other species, such as chimpanzees (*Pan troglodytes verus*) and green monkeys (*Cercopithecus sabaeus*), may spend less time searching and more time feeding on larger quantities of less nutritious food [7], [8]. In turn, these trade-offs will influence time available for other activities, such as defense and reproduction [9], which might then influence factors such as reproductive success [10].

Seasonal factors have been found to influence crop-raiding propensity by primates. From Sumatra and Sulawesi, two different macaque species were found to crop-raiding more frequently during wetter months [11], [12], which in turn was related to seasonal ripening of crops and low human activity on farms. Other determinants of primate crop-raiding occur within species, such as males exhibiting more risk prone behavior than females. From Bossou, West Africa, male chimpanzees (*Pan troglodytes verus*) were found to take greater risks (ie. raiding crops in exposed areas near villages) in order to obtain cultivated fruits [13].

Amongst primates, great apes are particularly threatened by habitat alterations because of their relatively large home range sizes, high daily calorific requirements and complex social systems. Whilst information is available on the behavioural ecology of African great apes living in disturbed forests [14], it is lacking for the two great apes in Asia, Sumatran and Bornean orangutans (*Pongo abelii* and *P. pygmaeus*), that are both highly threatened and currently experiencing rapid habitat transformation [15]. Recently, two studies focused on orangutan populations living in nonprimary forests [15], [16]. Despite this attention, no clear conclusions can be drawn on how orangutans modify their behaviour to anthropogenic influence as both studies recorded orangutan population densities through nest census only. However, Meijaard et al. [16] did speculate that bark and inner cambium from *Acacia mangium* is an import ‘fallback’ food source for the orangutans.

Understanding how wild orangutans living in a human-dominated habitat modify their behaviour and ecology compared with wild orangutans living under normal forest conditions is important for predicting their survival prospects. Thus, this paper aims to investigate, how an isolated Sumatran orangutan population adapts to living in a fragmented agroforest landscape. To address this, we determine the: i) various temporal parameters that best explain crop-raiding patterns; (ii) differences between male and female crop-raiding patterns; and, (iii) activity budgets and dietary breadth for both wild and cultivated foods.

## Methods

### Ethics Statement

All research protocols applied within this manuscript were assessed and approved by the University of Kent and the Indonesian Ministry of Forestry (permit # 1039/FRP/SM/V/2007 and # 2756/FRP/SM/XI/2008) and adhered to the Principles for the Ethical Treatment of Non-Human Primates and to Indonesian law.

### Study Area

Field data were collected between February 2007 and February 2009 in a 3,234 ha closed agroforest system in Batang Serangan (3°43'58.99″N, 98°11'41.99″E), North Sumatra, Indonesia (refer to [17] for map of the study area). The study area consists of approximately 1,350 smallholder farms intermixed with 2,784 ha of degraded natural forest and is bordered by commercial oil palm (*Elaeis guineensis*) plantations, human settlements (≥6,000 people) on most sides, another oil palm plantation (450 ha) in the centre and a river to the south and south west. The average distance of the smallholdings is ≥5 km from the nearest villages.

The habitat type in this region is best described as a ‘coarse grained’ mosaic habitat, with sizeable blocks of different habitat types directly adjacent to each other. For example, one farm may contain more rubber (*Hevea brasiliensis*) than degraded forest, while neighbouring farms may contain slightly more cultivated fruit crops (hereafter ‘cultivated fruit’) such as jackfruit, durian (*Durio zibethinus*), jengkol (*Archidendron pauciflorum*) and petai (*Parkia speciosa*) than degraded forest or a mixture of all three. The farms have no forested perimeters and therefore no distinct separation of degraded forest and cultivated fruit. For over 25 years, the orangutan population has been completely isolated from the nearest wild orangutan population located within the Leuser Ecosystem conservation area [18].

### Field Data Collection

#### Rainfall and food presence

Over 24 months, rainfall data were collected using a standard precipitation tube rain gauge from a single place (3°43'577″N, 98°11'455″E), within the study area, once in the morning (0600hrs) and once in the evening (1800hrs). Due to uneven rainfall patterns, the wet or dry seasons were not clearly defined and, so, low and high rainfall seasons were respectively calculated as months with ≤246 mm and ≥246 mm of rainfall (mean monthly rainfall for the study period being 246 mm).

Smallholder farms were monitored daily for cultivated and wild fruit availability by at least two observers. Fruits were recorded as ‘available’ on a particular farm if at least five tree species had fruits growing in 50% or more of their individual canopies [17].

#### Crop-raiding and crop damage enumeration

Nine enumerators, from local communities, were trained over four weeks in the use of orangutan crop damage and conflict mitigation datasheets, which were modified from those produced by the IUCN/SSC African Elephant Specialist Group for monitoring human-elephant conflict [19]. Fifty farms were chosen for intensive focal surveys based on a spread of crop-raiding frequencies identified in Year 1 and willingness of the farmer to participate in the study. An independent crop-raiding incident was used as the basic unit of measurement, whereby crop-raiding by an orangutan on the same farm on the same day was classified as a single event, irrespective of whether it raided more than once [20]. Crop damage (fruit remains and debarked trees) was measured through daily visits to focal farms by local enumerators. These 50 farms were checked twice in a week.

#### Behavioural observations

Prior to conducting this study, four months was spent identifying, following and habituating individual orangutans, which had never been studied before, and training three focal followers from the local community. During the study, 16 individual orangutans were identified, 8 adults, 5 adolescents and 3 new born infants (Table 1). Behavioural data were collected on the 8 adults and 5 adolescents using focal animal sampling techniques developed for studying orangutans [21].

**Table 1:** Demographics of the 16 individual orangutans identified and habituated during a 2-year study in Batang Serangan with follow times based on two field methods (≥3 hour follow days and nest to nest follow days).

Orangutan	Sex/Age	Relationship	≥3 hrs	Nest - nest	Average ≥3 hrs
identity			days (+ total hrs)	days (+ total hrs)	hrs (±SD)
OU 1#	♂ Adult (F)	Unknown	34 (285)	12 (123)	8.4 (±2.8)
OU 2#	♀ Adult	Mother of OU 3	43 (380)	23 (196)	8.8 (±2.7)
OU 3†	♂ Infant		0 (0)	0 (0)	0 (0)
OU 4#	♂ Adult (U)	Unknown	48 (453)	29 (305)	9.4 (±2.7)
OU 5#	♀ Adult	Mother of OU 6	55 (532)	38 (397)	9.7 (±2.4)
OU 6#	♀ Adolescent		55 (540)	38 (356)	9.8 (±2.6)
OU 7#	♀ Adult	Mother of OU 8	16 (162)	13 (134)	10.2 (±2.5)
OU 8#	♂ Adolescent		16 (161)	13 (135)	10.1 (±2.5)
OU 9#	♂ Adult (F)	Unknown	26 (259)	15 (157)	10.0 (±1.8)
OU 10#	♀ Adult	Mother of OU 11 & 12	22 (170)	11 (94)	7.7 (±3.4)
OU 11#	♀ Adolescent		22 (167)	11 (98)	7.6 (±2.9)
OU 12†	♀ Infant		0 (0)	0 (0)	0 (0)
OU 13#	♀ Adult	Mother of OU 14 & 15	29 (249)	16 (148)	8.6 (±2.9)
OU 14#	♂ Adolescent		29 (267)	16 (157)	9.2 (±2.9)
OU 15†	♀ Infant		0 (0)	0 (0)	0 (0)
OU 16#	♀ Adolescent	Unknown	3 (14)	0 (0)	4.5.(±1.0)
Total			398 (3639)	235 (2300)	
Shortfall			163 follow days + 1339 follow hours		

F  =  flanged adult male and U  =  unflanged adult male.

#denote crop-raiding and orangutans used in activity budget analysis, †denote born during the study period.

Individual orangutans were typically located by searching randomly in the farms on a daily basis. The three followers also used information provided by local farmers on their recent orangutan sightings. Once a focal orangutan was encountered in the field, the followers undertook, when possible, nest-to-nest follows and recorded the individual's behaviour using instantaneous point sampling. Follows were continued whenever possible for a maximum of five consecutive days, unless the individual was lost prior to this. During all follows, four main activities were noted at two minute intervals; travelling, resting, feeding and other (i.e. social behaviour). For feeding, wild fruits and cultivated fruits were recorded, and identified as fruit, leaves (further differentiating between young and old leaves), seeds (with no flesh consumed), bark, and branch (comprising fibers; [21]). A total of 706 days were spent searching for orangutans, from which they were encountered and followed on 398 days (≥3 hours duration), yielding a total of 3,639 follow hours (Table 1). Orangutans were encountered during each study month except January (Year 2).

The standard method used for measuring orangutan ‘active period length’ is to begin recording once an orangutan first sits up in its morning nest until it lays down in its night nest [22]. For this study, calculating the active period length from nest to nest follow days only would have markedly reduced the dataset (i.e. from 3,639 to 2,300 follow hours), as orangutans were often first encountered after they had left their morning nest or lost after several follow hours. To address these limitations, nest-to-nest (i.e. full day follows) and minimum follow limits ≥3 hours [23] were both used in the statistical analyses.

### Data Analysis

Orangutan activity budget and feeding behaviour data were imported into SPSS v.16.0 (Chicago, USA). When necessary, continuous data were normalised to reduce the disproportionate influence of outliers. Possible differences in inter-annual patterns of crop-raiding, rainfall, and fruit production were examined by sub-dividing the study period as Year 1 (February 2007-January 2008) and Year 2 (March 2008-February 2009).

#### Temporal crop-raiding patterns

Monthly crop-raiding patterns for each of the 50 focal farms were determined by calculating the mean crop-raiding incidents per month and per farm (referred to hereafter as ‘crop-raiding frequency’). Seasonal patterns of raiding on the main cultivated fruit species were examined using a Spearman's rank correlation coefficient (r_s_) to compare crop-raiding frequencies with mean daily rainfall patterns per month, with and without a 1-month lag period (as some crops may ripen after the rains). Rainfall may have significant impacts on the abundance of cultivate fruits across the landscape. Analysing crop-raiding patterns with rainfall patterns, directly and with a 1-month lag phase, allows us to fully examine correlation difference between two phases.

#### Behavioural observations

The monthly proportion of activity budgets and dietary compositions were determined for each individual orangutan by calculating the proportion of feeding time each month that was spent eating food item *i* and the proportion of follow time each month spent in activity *i*. MANOVA and Linear mixed-effect models were used to investigate differences in orangutan activity budgets between months of high and low rainfall, between study years, between the sexes and between crop-raiding and non-crop-raiding days. ONE WAY ANOVA was used to test if there was a shift in diets between different parts of the day, i.e. morning (0600–1100 hrs), afternoon (1100–1600 hrs) and evening (1600–2100 hrs), between study years and mean month time spent eating cultivated and wild fruits.

During data analysis, each follow day was classified either as a crop-raiding day, when orangutans ate cultivated fruits, or a non crop-raiding day, when orangutans ate only wild fruits. Chi-squared test was performed to investigate differences between orangutan crop-raiding days between study years.

## Results

### General features of orangutan crop-raiding patterns

During 398 follow days, orangutans were observed eating both cultivated (including bark) and wild fruits on 152 crop-raiding days, and wild fruits only on 74 non crop-raiding days with significantly more crop-raiding days in Year 1 (n = 106 days) than Year 2 (n = 46 days; χ^2^ = 24.291, df = 1, P<0.001). Orangutans ate only cultivated fruits, on 10 of these 152 crop-raiding days. From the 16 orangutans identified, all of the three adult males (2 flanged and 1 unflanged), five adult females and five adolescents were recorded crop-raiding.

### Temporal patterns

The number of wild and cultivated species available were higher in Year 1 (n = 27 wild fruit species and n = 9 cultivated fruit species) than in Year 2 (n = 23 wild fruit species and n = 6 cultivated fruit species). The number of wild fruits available was not correlated with rainfall patterns in Year 1 (r_s_ = 0.113, P = 0.727) but was negatively correlated with mean daily rainfall per month in Year 2 (r_s_ = −0.577, P<0.05). Conversely, the number of cultivated fruits available was positively correlated with rainfall in Year 1 (r_s_ = 0.577, 273 P<0.05) but not in Year 2 (r_s_ = 0.503, P = 0.095). The number of both wild and cultivated fruits per month (n = 24) did not correlate with overall temporal patterns in crop-raiding (r_s_ = 0.252, P = 0.235), but increased availability of certain cultivated fruits resulted in increased crop-raiding (jackfruit: r_s_ = 0.564, P<0.01; and, durian: r_s_ = 0.526, P<0.05).

Orangutan feeding habits differed between years (Table 2). During Year 1, jackfruit and durian fruits were raided more frequently in the wetter months (as indicated by a positive correlation with rainfall). Conversely, when these and other highly nutritious fruits were not available (i.e. during the drier months), the bark of jackfruit and rubber trees was raided significantly more, indicating its importance as a fallback food. In contrast, little fruit was available in Year 2, especially of the key cultivated fruit species (jackfruit and durian) and no temporal association was found.

**Table 2:** Relationship between mean monthly crop-raiding frequency and mean daily rainfall patterns per month based on Spearman's rank correlation coefficient (r_s_) with and without a one month lag phase.

	Year 1		Year 2	
	Frequency		Frequency	
	No lag	Lag	No lag	Lag
Crop species				
Jackfruit	0.52	0.56	0.12	−0.25
Durian fruit	0.64*	0.36	−0.39	0.13
Jengkol fruit	0.04	−0.53	−0.36	−0.80**
Petai fruit	−0.09	−0.23	0.30	−0.10
Jackfruit tree bark	−0.66*	−0.10	0.60*	0.41
Rubber tree bark	−0.09	−0.38	−0.16	−0.62*

*<0.05,

**<0.01.

### Sex differences in crop-raiding patterns

Linear mixed-effect models revealed that crop-raiding propensity by individual orangutan was significantly influenced by sex class (F_1,89_ = 4.117, P<0.05) and study years (F_1,89_ = 13.337, P<0.01), but not by rainfall (F_1,89_ = 0.015, P = 0.903). Overall, female orangutans raided agricultural fruits (115 separate feeding events; mean±SE; 4.8±0.2) more than male orangutans (on 82 separate feeding events; 3.4±0.3) and in the first year of the study, with no such differences in the second year. There was, however no significant sex-related differences in the mean time spent feeding on cultivated fruits and bark (F_1,325_ = 2.181, P = 0.141), as females spent a monthly average of 33.8 minutes (SD±38.4) and males of 43.7±56.5 minutes. There was no significant difference in mean time related to fruit and bark preference between the sexes (F_1,325_ = 0.487, P = 0.486), nor was there any evidence of seasonal preference (i.e. mean time spend crop-raiding during high and low rainfall months) between the sexes (F_1,325_ = 2.045, P = 0.154).

**Figure 1:**
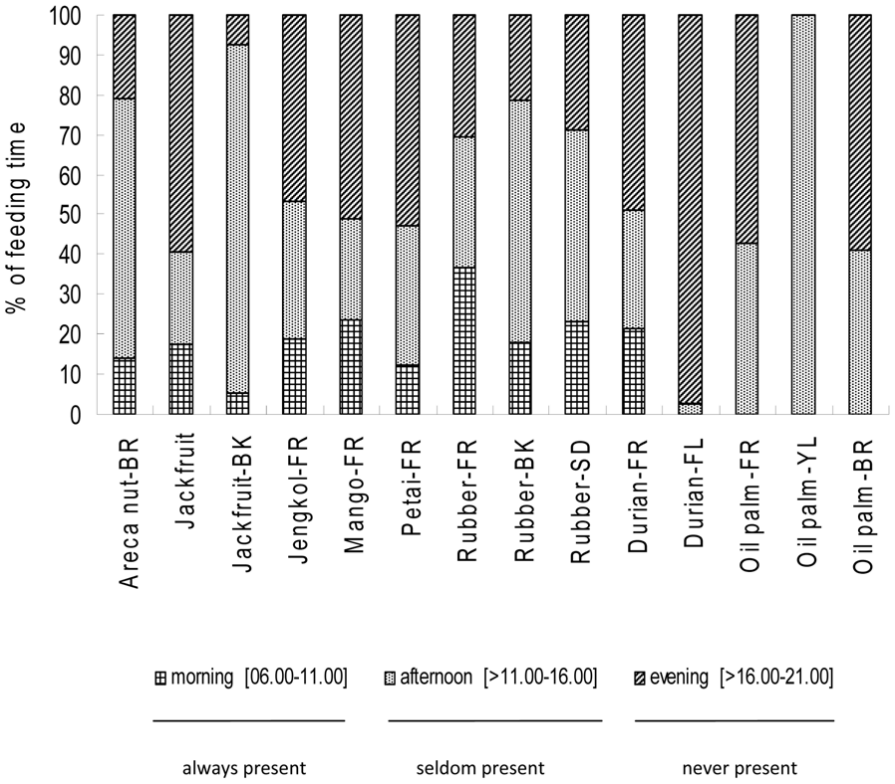
Observed patterns of eating and debarking of cultivated fruits during and whether there are farmers presence or not during each time zone. (BK  =  bark; BR  =  branch; FL  =  flower; FR  =  fruit; YL  =  young leaf; SD  =  seed.)

The average daily time spent feeding on cultivated fruits and bark differed across the three time periods (ANOVA, F_1,300_ = 7.031, P<0.01; Fig. 1). Tukey's HSD post-hoc tests, reveals significant differences found for morning-afternoon (P<0.05) and morning-evening (P<0.05), but not afternoon-evening (P = 0.870). Orangutans spent less time feeding on cultivated fruits and bark in the morning (17%) than in the afternoon (41%) and evening (42%). Fruits, with the exception of rubber, were predominately (49%) raided in the evening, whereas debarking of cultivated trees mainly (74%) occurred in the afternoon. These patterns did not significantly differ over the two study years (ANOVA, F_1,300_ = 2.336, P = 0.127).

### Consumption of wild and cultivated fruits

Feeding time on both wild and cultivated fruits combined over both study years accounted for the largest part (mean = 46%; range = 0–78%) of the orangutan population feeding budgets, than bark (33%; 5–94%), leaves (13%; 0–16%), seeds (7%; 0–16%) or other food products (flowers and insects, 1%; 0–10%; Fig. S1).

There were significant differences found between the orangutan population feeding budgets between study years (MANOVA, F_1,22_ = 4.068, P<0.05; Wilk's λ = 0.7894) but not between seasons (MANOVA, F_1,22_ = 1.556; P = 0.226; Wilk's λ = 0.686). Tukey's HSD post-hoc tests, reveals a significant difference in fruits (P<0.05) and bark (P<0.05) consumption between the study years. Overall, orangutans devoted most feeding time in Year 1 to eating fruits, both wild and cultivated representing 56% of all feeding observations (range = 17–78%). In contrast, orangutans devoted most feeding time in Year 2 to eating bark (44%; 8–94%).

Of the 3,639 follow hours, orangutans spent less time each month eating cultivated fruits, including bark, than wild fruits and bark (ANOVA, F_1,45_ = 12.994, P<0.001). Overall raided fruits and tree bark contributed to 21% (mean minutes/month±SE, 43.9±20.5) of the orangutan populations total feeding time, whereas wild species, including bark, contributed to 79% of the total feeding time.

### Activity budgets

Orangutans spent most of the observation time resting (mean = 54%; range = 11–81%), followed by feeding (24%; 8–59%), travelling (15%; 7–38%) and other activities (7%; 0–18%) such as nest building and mating (Fig. S2). There were no significant differences found between the orangutan population activity budgets (i.e. travel, rest, eat and other activity) between sex classes (MANOVA, F_1,58_ = 0.568, P = 0.687; Wilk's λ = 0.942), on days that orangutans were observed to crop-raid (MANOVA, F_1,58_ = 1.464, P = 0.226; Wilk's λ = 0.902) or during the months of high and low rainfall (MANOVA, F_1,22_ = 0.020, P = 0.384; Wilk's λ = 0.793). There was however, a difference between the study years (MANOVA, F_1,22_ = 7.198, P<0.001; Wilk's λ = 0.371). Tukey's HSD post-hoc tests, reveals a significant difference in ‘other’ activities between both study years (P<0.001). Overall, the orangutan population spent significantly more percentage of their activity budget resting (54%) than on other behaviour, such as feeding (24%).

## Discussion

As the first temporal study of an isolated orangutan population living in an agroforest system with no periodic access to natural forests, our results indicate that as a coping mechanism orangutans have modified their behaviour to living in a human-dominated landscape. This was illustrated, for example, by orangutans altering their diets as 21% of their total feeding activity budget was on cultivated fruits and by changing their foraging behaviour to raiding crops in the late afternoon or evening, which is when almost all farmers had returned to the village for the night. Thus, the future of this orangutan population will strongly depend upon the maintenance of natural forest food sources, otherwise conflicts with farmers are predicted to greatly increase.

### Sex of crop-raiders

Comparing between the sexes revealed that female orangutans were, on average, more likely to raid crops than males. This result differs from those found by studies of other polygynous primate species, e.g. chimpanzees [13] and vervet monkeys [24], where males were recorded taking greater crop-raiding risks. For adult male orangutans seeking to maximize their nutritional intake and, in turn, body mass to achieve social dominance and ultimately increased reproductive success, similar findings would have been expected from Batang Serangan. However, there are several plausible reasons as to why this was not the case. First, intrasexual competition between males was probably lower than that found in larger populations of forest-dwelling primates, as there were only three adult males in this study area, of which one (OU1; Table 1) was visibly much larger, had won direct combats with both of the other males (pers. obs.) and, therefore, achieved dominance over them. Second, the risk posed to crop-raiding individuals, of either sex, was probably lower than in other studies because the Batang Serangan farmers rarely retaliated if they encountered a crop-raiding orangutan [18] and these forays were typically conducted in the farmers' absence. Thus, females, especially those with newborns and therefore at greater risk, would be at no discernable disadvantage than males when crop-raiding.

### Dietary diversity and temporal foraging patterns

**Figure 2:**
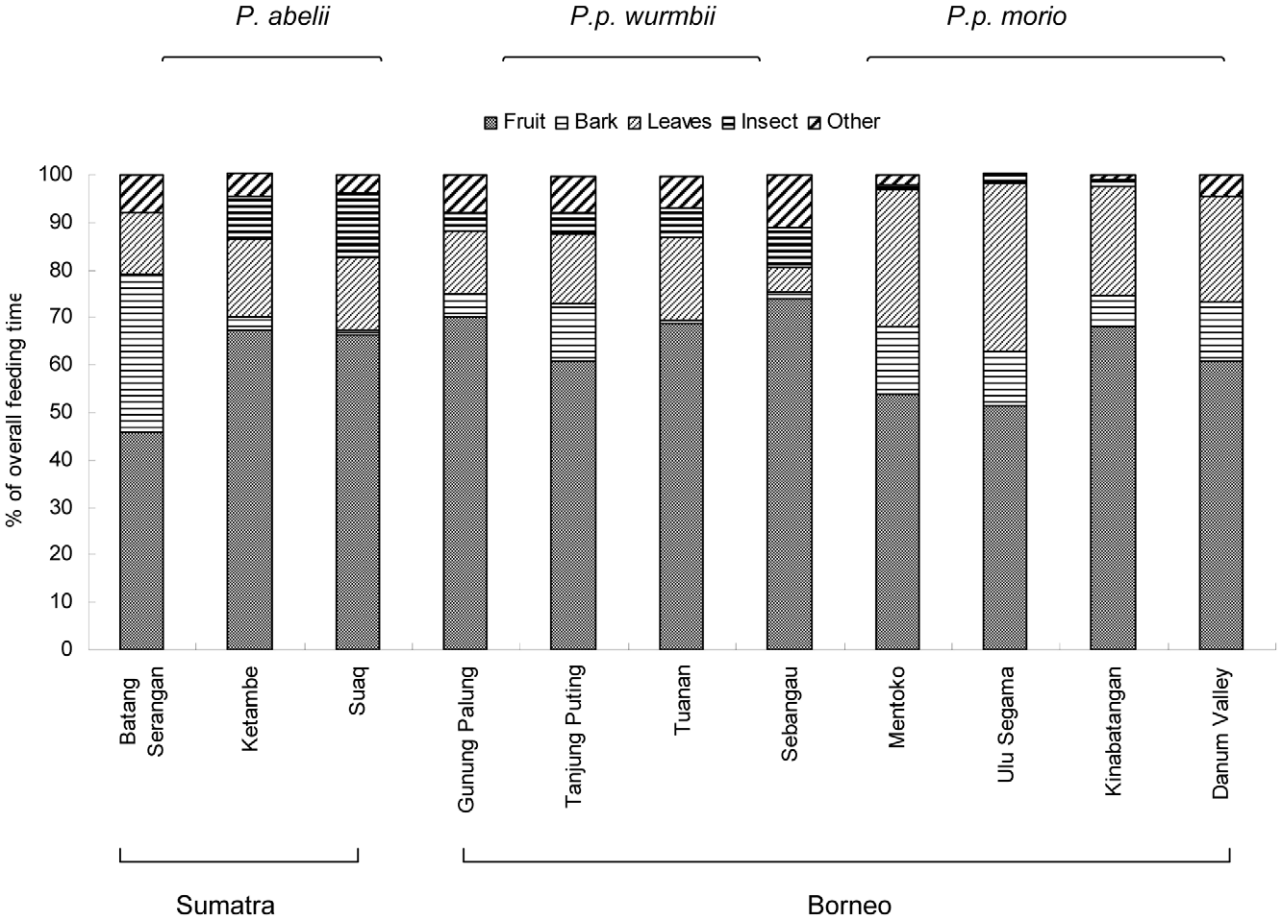
Percentage of overall feeding time on each food category at Batang Serangan, compared with ten other sites. Data adapted from the following [22], [40].

In Batang Serangan, orangutan diet consisted of 79% wild fruits and leaves, suggesting that cultivated fruits supplemented daily food intake. Long-term orangutan studies from Sumatra and Borneo also show high levels of fruit and leaf intake [22], [25], [26], [27]. In two Sumatran primary forest research sites (Suaq Balimbing and Ketambe), the percentage of time orangutans fed on fruits was 66–68% of their activity budget, 16% eating leaves, and 1–3% eating bark [22], [25], [28]. In comparison, orangutans in the agroforest landscape spent less percentage of time eating fruits (46%), a similar (13%) percentage of time eating leaves and more percentage of time (33%) eating bark than wild Sumatran orangutans in Suaq Balimbing and Ketambe (Fig. 2). By contrast, orangutans at three Bornean sites spent an average of 11–14% of their feeding time on bark. Bornean orangutans have also be recorded as allocating 31–67% of their time feeding on bark [22], [28]. However these were monthly maximum values and not the average total feeding time.

Orangutans in Batang Serangan allocated more time and consumed more bark than reported from elsewhere, which may reflect the greater habitat disturbance, as this agroforest system closely resembles disturbed forest habitats on Borneo, where orangutans also depend on bark as a fallback food [22]. More species of wild and cultivated fruits were available in Year 1 than Year 2, when bark was more frequently raided. In contrast, chimpanzees (*Pan troglodytes verus*) at Bossou fed more on crops when wild fruits were scarce [29], the crop-raiding frequency of orangutans in this study was high when cultivated and wild fruit were most available. Therefore, crop-raiding levels were best explained by the availability of ripe cultivated fruits rather than a scarcity of wild fruits [30], [31].

Overall crop-raiding frequencies were higher for the five main crop species during the fruiting seasons, which concurred with reports by local farmers [18]. Such selectivity may be related to crop/tree abundance, after rubber, jackfruit was the most commonly cultivated species within the farms. Similarly, Hill [32] suggested that some crops will attract more damage simply because they are grown at higher quantities. A study on crop-raiding gelada baboons in Ethiopia and vervet monkeys in Uganda also showed that crops most commonly cultivated were reported as the most damaged [33], [34] .

Our results from Year 1 concurred with other published studies that orangutans are primarily frugivorous. However, orangutans spent more (44%) time eating bark than fruit (35%) in Year 2, when irregular fruiting patterns may have forced orangutans to find alternative food resources that were less nutritious. When relying heavily on such fallback foods in Year 2, it might be predicted that orangutans would allocate a greater percentage of their activity budget to feeding in order to meet energetic and nutritional requirements. However, this was found not to be the case, as discussed further below.

### Activity budgets

**Figure 3:**
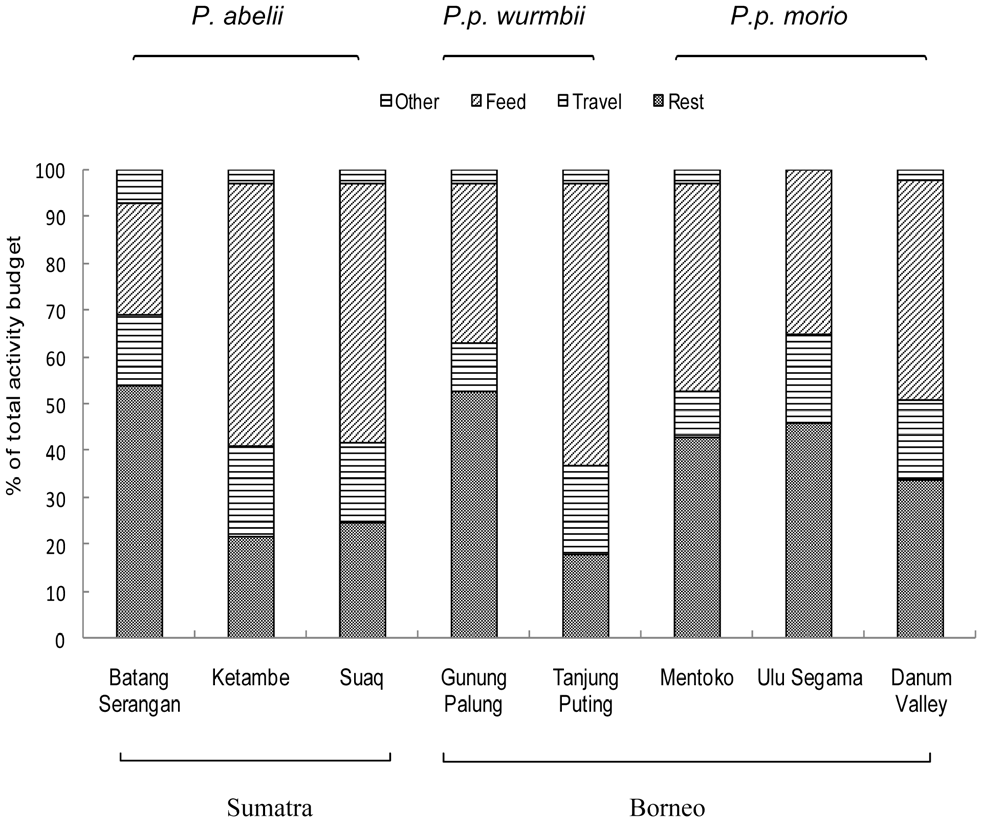
Percentage of overall time spent in each activity category at Batang Serangan, compared with seven other sites. Data adapted from the following [22], [25], [27], [40], [41], [42], [43].

Overall, the orangutan population spent significantly more percentage of their activity budget resting (54%) than on other behaviour, such as feeding (24%). The results contrast with those found in primary forest dwelling Sumatran orangutans [22], [25], which spend more time feeding (55%) than resting (25%, Fig. 3). The activity budgets at Batang Serangan are similar to Bornean orangutans living in forest with irregular fruiting patterns or that have been logged [22]. The orangutan activity budgets from this human-dominated landscape are similar to those found in other primate crop-raiding studies. From Kenya, semi-provisioned baboons (*Papio cynocephalus*) that fed from a food waste dump spent more time resting and socializing and less time feeding compared to an adjacent wild group [5]. Likewise, the consumption of human food had a pervasive influence on the activity budget of a crop- and food-raiding group of free-ranging vervets (*Chlorocebus aethiops pygerthrus*), which spent more time resting than feeding [34].

Optimality models [9] would suggest that orangutans in disturbed habitats would adapt by either expanding their dietary breadth to spend longer feeding bouts on less favoured fallback food items, or increase their search time to seek out and consume more nutritious, high energy foods. Thus, the inclusion of high energy cultivated fruits (e.g. durian = 147 kcal and 27 g carbohydrates per 100 g; jackfruit  = 94 kcal and 24 g carbohydrate per 100 g; [35]) would allow for these orangutans to meet their metabolic needs sooner than under natural forest conditions.

Orangutans can adapt to regular fluctuations in food availability [36], by altering their foraging strategies and diets as a coping mechanism [37]. Therefore it is not unexpected that the animals in this study devoted a disproportionally large amount of their time to resting, perhaps in order to conserve daily energy output due to consuming higher than normal lower quality fallback foods such as bark (33% consumed in their overall dietary breadth), of which caloric content is less than that of fruits [38]. However, the time allocated to feeding did not change between our two study years even though an increased reliance on fallback foods was recorded. Thus, the ‘sit-and-wait’ strategy adopted by orangutans is similar to that found in other crop-raiding primates [5], [22], [39].

Our study reveals that the orangutan population does not entirely rely upon cultivated fruits as their main food source, indeed 79% of the population's diet consisted of wild fruits. When orangutans did crop-raid, the farmers did not report this species to be the main threat to their livelihoods, instead they correctly blamed Thomas' leaf monkeys [18]. This study reveals that orangutans can coexist with people by ecologically adapting to the varying costs and benefits presented by the agroforest system that has been heavily degraded [17]. Taken together, this suggests that mitigating crop-raiding on the farmers' most valued cash crops based on the temporal patterns of crop-raiding identified in this study would be the logical next step. Future investigations might also incorporate more detailed nutritional analyses of both wild and cultivated fruits including new introduced foods and fallback foods, in order to further assess orangutan behavioural ecology.

## Supporting Information

Figure S1 **Monthly orangutan diet composition including cultivated and wild fruit species expressed as a percentage of overall feeding time.**
(PDF)

Figure S2 **Monthly orangutan activity budgets expressed as percentage of overall activity budget.** Social, nest building and playing were also recorded separately but later categorized as ‘Other.’
(PDF)
