# Supplementary figures and images for: Raiders of the Lost Bark: Orangutan Foraging Strategies in a Degraded Landscape
Source: PLoS One. 2011 Jun 22;6(6):e20962. doi: 10.1371/journal.pone.0020962 (PMC3120831; doi:10.1371/journal.pone.0020962)

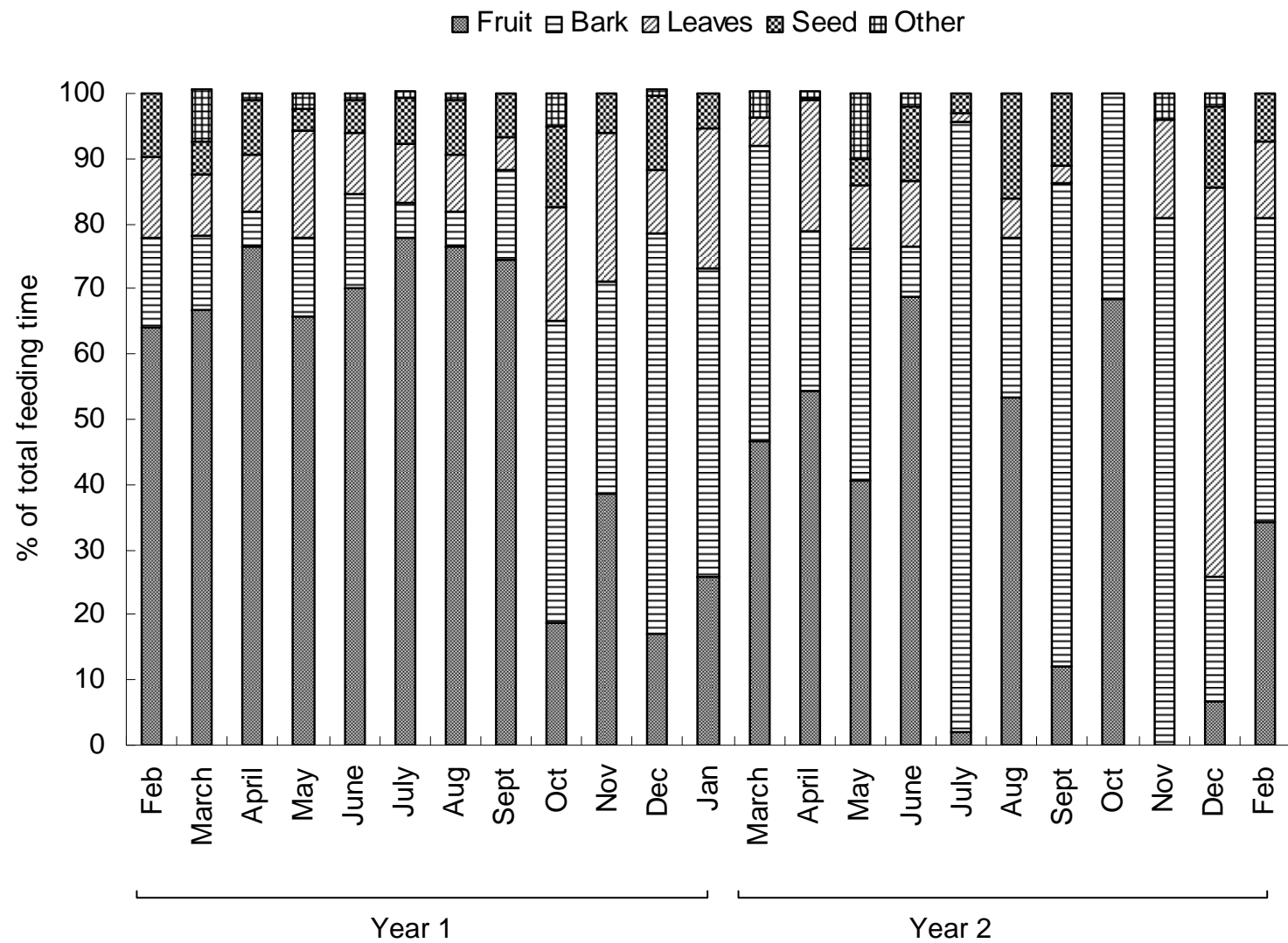

Supplement: Figure S1 — Monthly orangutan diet composition including cultivated and wild fruit species expressed as a percentage of overall feeding time. (PDF) [file pone.0020962.s001.pdf]

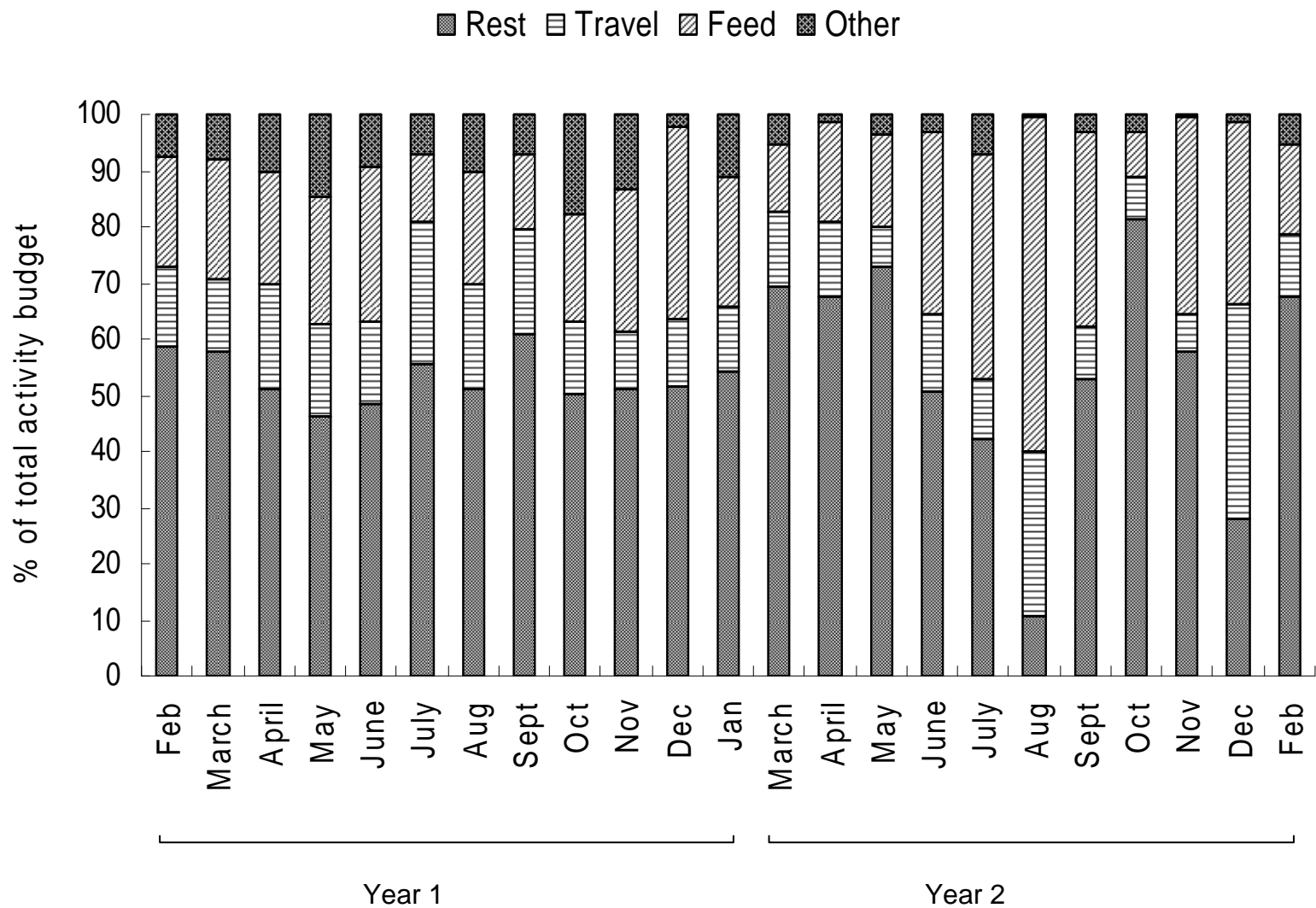

Supplement: Figure S2 — Monthly orangutan activity budgets expressed as percentage of overall activity budget. Social, nest building and playing were also recorded separately but later categorized as ‘Other.’ (PDF) [file pone.0020962.s002.pdf]
